# Diagnostic Value of Muscle Ultrasound for Myopathies and Myositis

**DOI:** 10.1007/s11926-020-00947-y

**Published:** 2020-09-28

**Authors:** Jemima Albayda, Nens van Alfen

**Affiliations:** 1 School of Medicine, Division of Rheumatology, Johns Hopkins University, Baltimore, MD USA; 2 Department of Neurology and Clinical Neurophysiology, Donders Institute for Brain, Cognition and Behavior, Radboud University Medical Center, Nijmegen, The Netherlands

**Keywords:** Muscle ultrasound, Myopathies, Myositis, Diagnostic test, Muscle echogenicity

## Abstract

**Purpose of Review:**

The purpose of this review is to critically discuss the use of ultrasound in the evaluation of muscle disorders with a particular focus on the emerging use in inflammatory myopathies.

**Recent Findings:**

In myopathies, pathologic muscle shows an increase in echogenicity. Muscle echogenicity can be assessed visually, semi-quantitatively, or quantitatively using grayscale analysis. The involvement of specific muscle groups and the pattern of increase in echogenicity can further point to specific diseases. In pediatric neuromuscular disorders, the value of muscle ultrasound for screening and diagnosis is well-established. It has also been found to be a responsive measure of disease change in muscular dystrophies. In chronic forms of myositis like inclusion body myositis, ultrasound is very suitable for detecting markedly increased echogenicity and atrophy in affected muscles. Acute cases of muscle edema show only a mild increase in echogenicity, which can also reverse with successful treatment.

**Summary:**

Muscle ultrasound is an important imaging modality that is highly adaptable to study various muscle conditions. Although its diagnostic value for neuromuscular disorders is high, the evidence in myositis has only begun to accrue in earnest. Further systematic studies are needed, especially in its role for detecting muscle edema.

## Introduction

The evaluation of muscle disorders and myopathies is greatly enhanced by the addition of imaging to identify structural abnormalities and provide an assessment of muscle quality. Ultrasound (US) of muscle was first described in 1968 [1], making it one of the first imaging modalities applied to muscle. The advent of improved technology has led to higher resolution sonography for soft tissue evaluation that is very suitable for muscles. Additionally, the patient-friendly nature, the lack of contraindications, and the ability to assess muscles dynamically in real time and at the bedside are important advantages of this tool. In this review, we will discuss the applications of muscle US for the diagnosis and evaluation of myopathies with a special focus on myositis.

## Muscle Sonoanatomy

### Normal Muscle

**Fig. 1:**
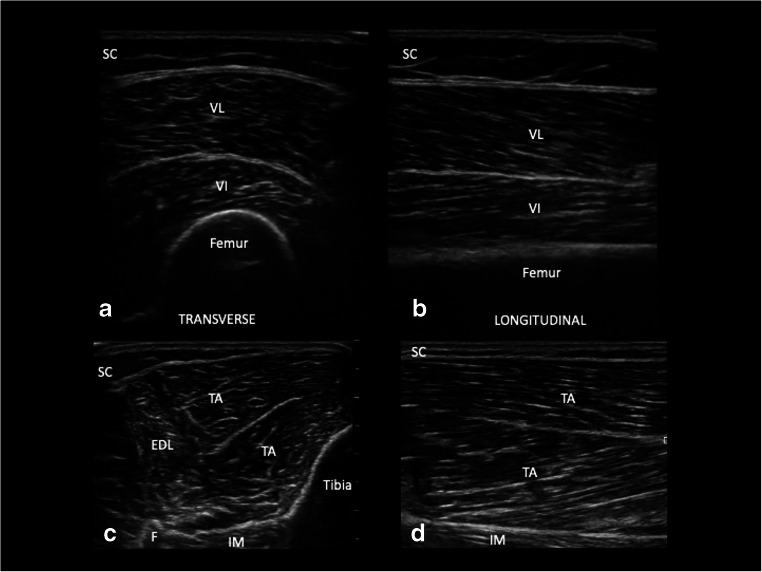
A normal vastus lateralis (A, B) and tibialis anterior (C, D) in transverse and longitudinal views. SC, subcutaneous tissue; VL, vastus lateralis; VI, vastus intermedius; TA, tibialis anterior; EDL, extensor digitorum longus; IM, interosseous membrane

On cross-section, normal muscle appears as a relatively anechoic structure with hyperechoic speckles within the tissue representing perimysial septa, giving it the “starry night appearance” (Fig. 1A, C). The boundaries of each muscle are delineated by the presence of hyperechoic fascia. On longitudinal view, the parallel orientation of muscle fibers is appreciated as well as the angle by which it inserts onto bone or an aponeurosis (Fig. 1B, D). The appearance of muscles from each anatomic location is different on US and is dependent on the ratio of contractile elements to connective tissue, muscle size and structure. Additionally, muscle composition varies with age and sex [2].

A particular property of muscles and tendons is anisotropy. In tissues that have colinear structures, the direction of sound reflection will change uniformly depending on the angle of insonation [3]. This has particular implications for imaging muscle as the echogenicity of the tissue can change with slight angulations of the probe. To aid in finding the maximal angle at which the muscle is imaged perpendicularly, structures such as bone or the deep fascia are used as a guide with the images captured at the angle where bone or fascia is most distinct [4].

Other parameters that can be assessed on muscle US include muscle thickness, cross-sectional area, fascicle length, and pennation angle [1, 5–7]. These structural parameters have been shown to provide information about muscle strength and correlate well with strength measurements.

### Pathologic Muscle

In muscle disorders, one of the hallmark findings is replacement of healthy muscle with fat and fibrosis, manifested by an increase in echogenicity from higher sound transitions in the muscle [8]. This increase in echogenicity is most distinct in conditions that lead to chronic pathology, such as long-standing muscle inflammation, dystrophy, or denervation [9].

**Fig. 2:**
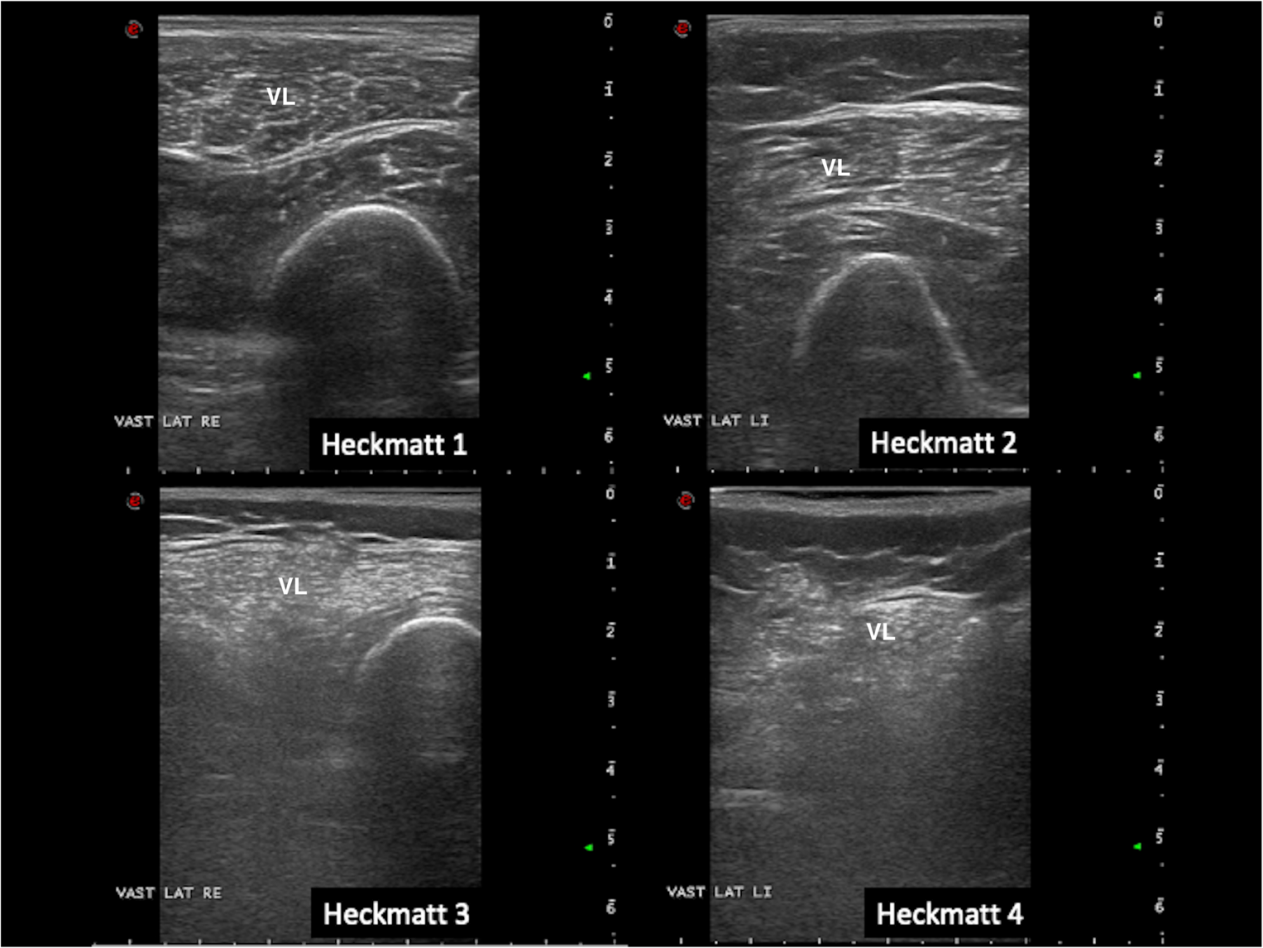
Heckmatt scoring using vastus lateralis muscle (disease examples from established myositis patients); grade 1 is normal; grade 2 shows a slight increase in echogenicity without architecture loss or attenuation. Grade 3 shows clearly increased muscle echogenicity, loss of muscle architecture, and some attenuation causing less visibility of deeper structures. Grade 4 shows a completely white muscle with loss of recognizable features and strong attenuation of the ultrasound signal so no deep structures can be discerned beyond the superficial layer of muscle. VL, vastus lateralis

**Fig. 3:**
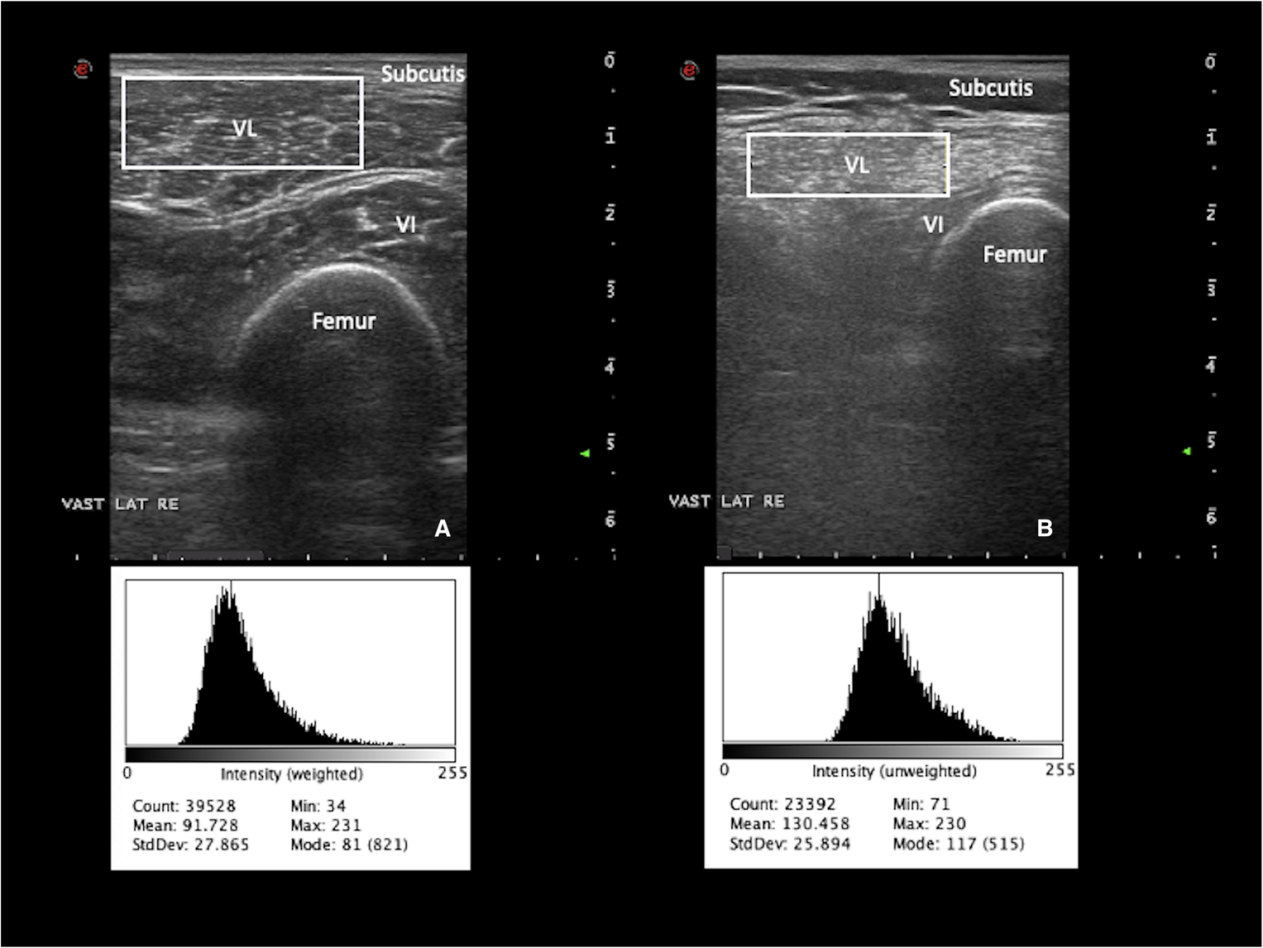
Quantitative grayscale analysis in a healthy (A) and diseased muscle (B). The mean echogenicity (EI) can be read from the histogram output using Image J (https://imagej.nih.gov/ij/). In (B), the muscle can be seen as having an increase in echogenicity compared with overlying subcutaneous tissue and normal muscle in (A). Mean grayscale level in the normal vastus lateralis is 91 (A) versus 130 in the diseased (B). VL, vastus lateralis; VI, vastus intermedius

Several methods can be used to assess muscle echogenicity. The first is a visual method based on evaluating echogenicity in relation to other structures such as subcutaneous tissue, which should be of similar echogenicity. The second is a semi-quantitative method based on a scale described by Heckmatt (1, normal muscle; 2, increase in muscle echogenicity with normal bone echo; 3, moderate increase in muscle echogenicity with decreased bone echo; 4, severe increase in muscle echogenicity with shadowing obscuring the underlying bone echo (Fig. 2)) [4]. The reported sensitivity for visual assessment for detection of myopathies is about 70% [10]. A third, quantitative method of assessing echogenicity is also available, with the use of grayscale analysis and reference values. Using image software capable of obtaining a region of interest in the muscle and utilizing histogram functions to ascertain the mean gray-level (0–255) in a region, muscle echogenicity can be easily quantified (Fig. 3). The use of this method requires that system presets for each machine are made (fixed settings, depth), and that normative values are established for each system to aid in interpretation of results. When properly carried out, this is the most sensitive measure of muscle echogenicity that can be followed over time, with reported detection rates of more than 90% overall for neuromuscular diseases [10]. The finding of increased echogenicity in muscles clinically affected by a particular disease is especially useful for diagnostic purposes.

## Use in Myopathies

Muscle US is currently used in two domains: for screening and diagnostic purposes in suspected neuromuscular disease, and as a surrogate biomarker for follow-up of these disorders. Its role and value in screening for neuromuscular pathology in children is well-established [11••, 12•]. The positive predictive value of a short quantified muscle US screening protocol for the presence of a neuromuscular disorder was found to be 91% [13••]. For adults, there are no specific studies that evaluate the technique as a general screening tool, but it is assumed that sensitivity and specificity are similar to those in children. Besides studies that looked at muscle US use as a general (yes/no) screening tool for any neuromuscular disorder, there is further research showing muscle US changes in tissue echogenicity and muscle texture across the age span in different specific neuromuscular disorders such as Duchenne muscular dystrophy (DMD), spinal muscular atrophy (SMA), Pompe disease, facioscapulohumeral dystrophy (FSHD), congenital myotonias, amyotrophic lateral sclerosis (ALS), and different forms of myositis [14–22]. In general, myopathic changes are characterized by granular fine increases in muscle echogenicity which are most pronounced in muscular dystrophies leading to a homogenous appearance of the muscle [23]. In neurogenic changes, the muscle appears more inhomogenous, with interspersed hyperechoic areas corresponding to atrophic fibers, and hypoechoic areas corresponding to normal or hypertrophic fibers. In long-standing neuromuscular disease, the differences between myopathic and neurogenic changes on US become less distinct. The diagnostic values of muscle US for all these established neuromuscular disorders is high, meaning that it is unlikely that any of the patients who have such a specific neuromuscular disorder will be missed.

In addition, US adds the possibility of dynamic screening of voluntary and involuntary muscle movements, that improves the detection of pathologic phenomena such as fasciculations with 30–50% compared with electromyography [10]. Muscle US can even detect fibrillations as a sign of denervation if the appropriate image settings are used [24]. Dynamic muscle US can also detect respiratory muscle involvement such as diaphragm weakness with very high sensitivity and specificity [25].

Muscle US has also been evaluated as a surrogate biomarker and a follow-up tool for different neuromuscular disorders. It was found to be a responsive measure of disease change in muscular dystrophies such as DMD and FSHD [26, 27]. US measures were significantly correlated to clinical change in DMD, while in an FSHD population, the echogenicity level on muscle US was the only parameter that showed a significant change over a 1-year follow-up period. Muscle US also showed changes over time in ALS patients, but less consistently so [28, 29].

### Pattern Recognition

**Fig. 4:**
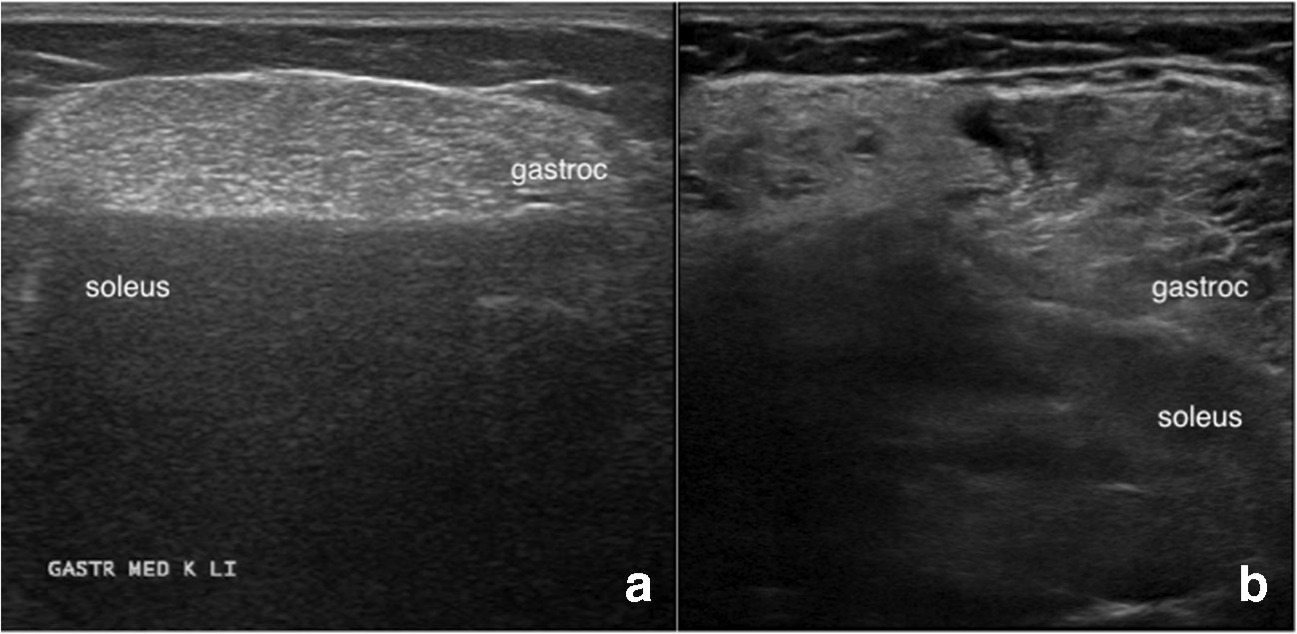
Increased echogenicity in a “ground glass” pattern (A), and a “moth-eaten” pattern (B)

**Fig. 5:**
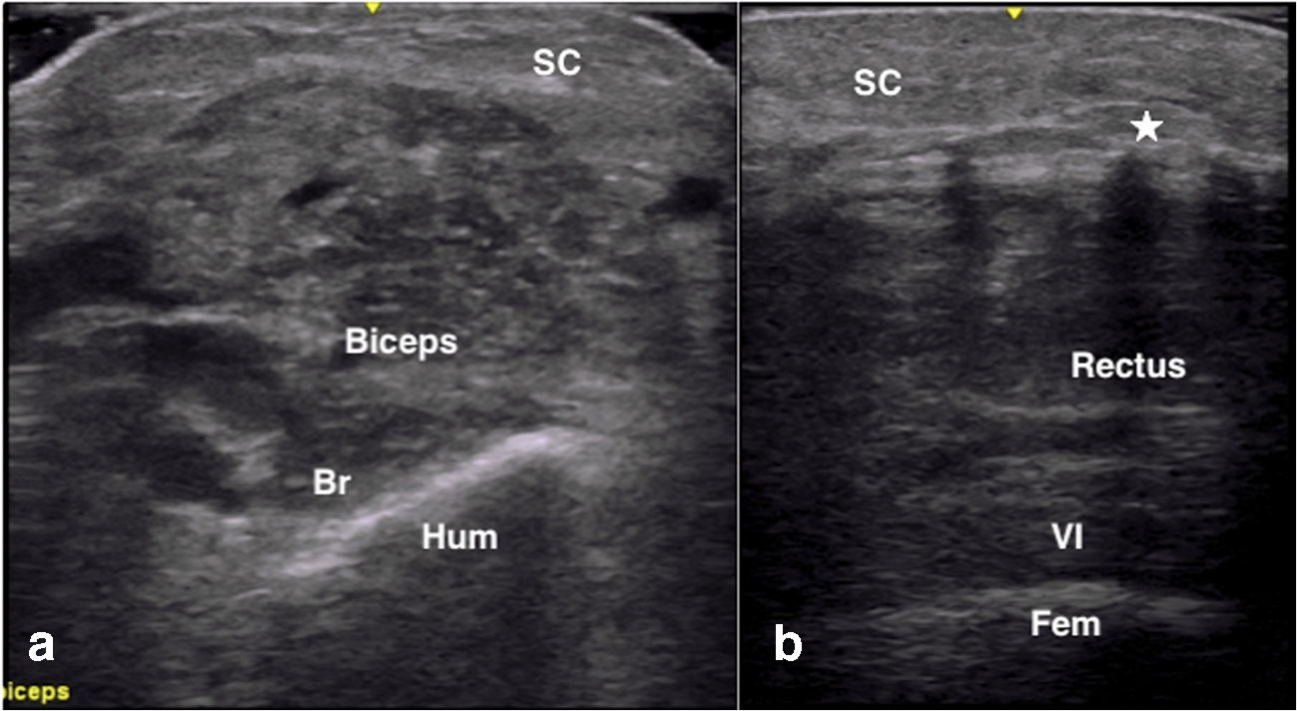
Dermatomyositis showing increased echogenicity in the subcutaneous tissue, focally altered and “see-through” echogenicity within the muscle (A), as well as calcinosis (*) causing posterior shadowing (B). SC, subcutaneous tissue; Br, brachialis; Hum, humerus; VI, vastus intermedius; Fem, femur

**Fig. 6:**
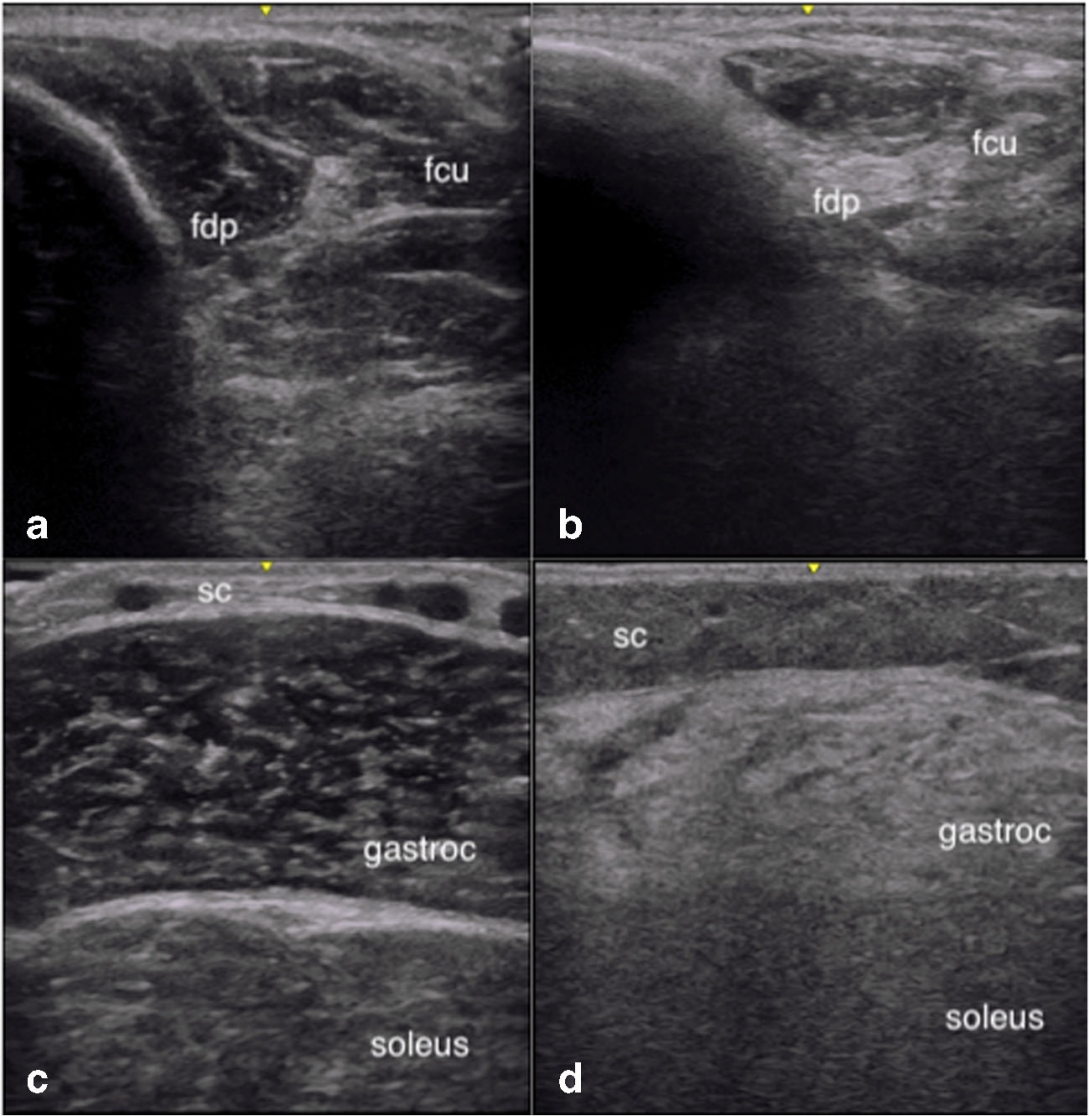
Affected flexor digitorum profundus FDP (B) and gastrocnemius (D) muscles in IBM showing markedly increase echogenicity and atrophy, in comparison with normal (A, C). fdp, flexor digitorum profundus; fcu, flexor carpi ulnaris; SC, subcutaneous tissue

Different types of muscle pathology lead to different muscle US appearances (Table 1). These patterns can inform the clinician on the type of disorder. Muscular dystrophies are associated with extensive replacement of muscle fibers by fat and fibrosis as the disease progresses. This results in a homogenously increased echogenicity of the entire muscle with loss of architectural features, dubbed as a “ground glass” or “rubbed out” (as in pencil marks by an eraser) appearance (Fig. 4A) [30]. Depending on the type of dystrophy, the muscle changes may be found throughout the whole muscle, or in specific regions only that expand over time (such as in FSHD) [31]. Atrophy is a variable feature in muscle dystrophies, and typically no atrophy is found in Duchenne muscular dystrophy, for example [16]. In inflammatory myopathies such as dermatomyositis and polymyositis, the abnormalities usually start out as focal areas of increased echogenicity (Fig. 5A) that spread with disease progression [32]. In acute disease, muscle edema can be seen as an overall echogenicity increase without attenuation of the underlying bone echo that has been dubbed a “shine-through” appearance or “see-through echogenicity increase” (Fig. 5). In chronic disease such as inclusion body myositis (IBM), atrophy as well as a markedly increased echogenicity is seen, oftentimes obscuring bone echo (Fig. 6) [33]. Inflammation of the skin and subcutis may also be found in dermatomyositis, together with calcinosis (Fig. 5B).

**Table 1:** Key sonographic findings in neuromuscular disorders

Neuromuscular disorder	Findings
Myopathies	Echogenicity often more abnormal than muscle thickness
Muscular dystrophies	Multifocal to homogenous increase in echogenicity with “ground glass appearance,” attenuation of muscle leading to normal or decreased echogenicity in deeper layers in severely affected muscles. Caveat: full fatty degeneration can give a falsely “normal” echogenicity, but muscle architecture will be lost
Congenital myopathies	Multifocal to homogenous increase in echogenicity, decreased muscle thickness, attenuation of muscle leading to normal or decreased echogenicity in deeper layers in severely affected muscles
Metabolic myopathies	Minimal changes on ultrasound, may have mild to moderate increase in echogenicity once structural changes become apparent
Inflammatory myopathies	In the acute phase, there is a slight increase in echogenicity. In the chronic phase, this becomes more pronounced and is accompanied by decreased muscle thickness. Focal increases in echogenicity can be seen. The muscle can return to normal appearance with successful treatment and remission.
Dermatomyositis	Echogenicity increase can be focal with a “see-through appearance,” accompanied by increased echogenicity of subcutaneous tissue
Inclusion body myositis	Markedly increased echogenicity of affected muscles and decreased muscle thickness. Muscles can have “moth-eaten appearance” and involvement can be asymmetric
Neuropathies	
Polyneuropathy	In mild neurogenic pathologies with reinnervation, the ultrasound appearance remains normal. In more severe cases with incomplete reinnervation, an inhomogenous increase in echogenicity and decreased muscle thickness is seen, in a “moth-eaten pattern.” Distal more severe than proximal muscles.
Focal neuropathy	First US abnormalities are visible after 10 days with echogenicity more abnormal than muscle thickness. With reinnervation, the ultrasound appearance remains normal. Areas without reinnervation will look “moth eaten.” Persistent denervation of the muscle will lead to markedly increased echogenicity, decreased muscle thickness with now black fascial lines.
Motor neuron disease	
Spinal muscular atrophy	Inhomogenous increase in echogenicity with “moth-eaten” appearance or completely white muscles with severe atrophy. Can be normal in infants with SMA type I.
Amyotrophic lateral sclerosis	Increased echointensity with decreased muscle thickness, but fasciculations are the most prominent feature.

Neurogenic disorders have a variable appearance on muscle US that depends on the severity of the axonal loss, the duration and progression of the disorder, and on whether reinnervation has occurred. Three patterns can be discerned. Usually no abnormalities are found in case of a slight monophasic axonal injury, such as a mild radiculopathy. In cases with longer standing denervation and incomplete reinnervation, muscle US will show a patchy to diffuse increase in echogenicity that can be found comparing the affected side with the contralateral side. The third pattern is the most characteristic and has been dubbed a “moth-eaten” pattern [30, 34]. It shows round, dark areas of viable motor units, surrounded by areas of increased echogenicity that reflects permanent denervation and fibrosis (Fig. 4B). This pattern is seen in long-standing and progressive neurogenic disease such as SMA and can also be found in long-standing IBM when fiber splitting has led to chronic denervation and reinnervation.

## Emerging Use in Myositis

The use of muscle US in the inflammatory myopathies has lagged behind its use in other myopathies, in part due to the more subtle changes of edema on US, which is better characterized on MRI. In one of the earliest studies investigating the myosonographic findings of myositis, histopathology was correlated with muscle US findings [22]. In those cases showing edema on biopsy, muscles were significantly less echogenic than those without edema, and had greater muscle thickness. In those with fat infiltration on biopsy, higher echogenicity and smaller muscles were noted compared to those without lipomatosis. Correspondingly, in cases of acute myositis (< 1 year), muscle size was usually normal and was accompanied by a relatively low echogenicity. Chronic myositis showed higher echogenicity and smaller muscles than acute cases. Likewise, other studies have shown an increased echogenicity in affected myositis muscle, including in cases of DM with normal muscle enzyme levels, suggesting it can be used to identify occult muscle disease [35].

In studies where follow-up was available, muscle US was responsive to changes induced by treatment. In a study of 7 juvenile DM patients evaluated at baseline, 1,3,6,12 and 24 months after initiating therapy, muscle echogenicity was only slightly increased and muscle thickness was relatively normal at baseline [36•]. After 3 months, the muscle thickness decreased and echogenicity further increased, followed by normalization of echogenicity within 6–12 months of treatment paralleling improvement on disease activity scores. In another study of adult patients where muscle US was completed at baseline and 6 months after treatment in 11 patients, they found that there was an increase in echogenicity in myositis patients compared with healthy controls, and these findings also improved with treatment [37]. This suggests that the acute phase of edema does raise echogenicity and this can be reversible with successful treatment.

US findings can also be an additional tool to augment an assessment of clinical disease activity. In a juvenile DM study that looked at patients with high disease activity (*n* = 7) versus those with low disease activity or were in remission (*n* = 10), muscle echogenicity, and not muscle thickness, could discriminate between high and low disease activity in JDM [38]. There was also a significant correlation between echogenicity, CPK and the Childhood Myositis Assessment Score. Fascial thickening has also been seen in both DM and PM using US, as well as an increased Doppler signal in fascia indicating fasciitis [39, 40].

For IBM where the lack of treatment and chronic changes in the muscle lead to a very notable increase in muscle echogenicity due to fibrosis and fatty replacement, muscle US has been very useful in evaluating for the disease. Affected muscles in IBM show a higher echogenicity and a lower muscle thickness which become more pronounced with longer duration of disease [21•, 41, 42]. The selective involvement of several muscle groups such as the flexor digitorum profundus (FDP), gastrocnemius, and quadriceps has been demonstrated to differentiate IBM from other similar diseases. A feature notable on visual assessment is the contrasting echogenicity between the affected muscle such as the FDP and gastrocnemius, and the adjacent healthy muscle of the flexor carpi ulnaris and soleus muscle, respectively (Fig. 6) [21•, 41]. A study using two cohorts showed that among these muscle groups, the FDP is the most discriminating muscle for IBM compared to myositis and neuromuscular mimics, followed by the quadriceps muscle when using the parameter of echogenicity [43•].

In terms of other connective tissue diseases, muscle US has also been used as a diagnostic and follow-up tool in patients with deep morphea, or morphea profunda [44, 45]. US of the skin and subdermis in areas with sclerotic plaques can show either inflammation with thickening or atrophy with thinning of the subcutaneous tissue layers, while muscle US will show hyperechogenicity and atrophy in cases with myositis [46]. In systemic lupus erythematosus, a study has been done to evaluate muscle changes in patients without a clinical diagnosis of myositis [47]. They found an increase in muscle thickness, pennation angle, and fascicle length in the vastus lateralis of lupus patients in comparison with controls. In contrast, a decrease in isokinetic knee muscle strength was seen, especially in those on steroids. The clinical significance of these findings is unclear, especially since there was no assessment of muscle echogenicity that may explain the qualitative changes in the muscle.

### Beyond Echogenicity: Other Techniques for Analyzing Muscle Ultrasound Images

Given some challenges surrounding the assessment of echogenicity in muscle, other techniques have also been applied to the analysis of muscle US images that do not take into account echogenicity alone. These have included texture analysis as well as machine learning methods [48–50, 51•, 52]. Deep learning methods are particularly interesting as they may provide more objective means for disease characterization that takes into account information from the whole image as opposed to the muscle alone. This may have implications in pattern recognition and automated diagnostics that can be useful for decreasing subjectivity and bias in interpretation, as well as for detecting edema more reliably.

Doppler has not been routinely used in the evaluation of myositis as it is a difficult parameter to standardize. Additionally, muscle has a low blood perfusion at rest. An earlier study looking at power Doppler in myositis found only mildly elevated vascularity scores (using a scale of 0–4) that did not reach statistical significance [53]. However it was noted that there was tendency to higher vascularity scores in patients with disease of shorter duration. Contrast-enhanced ultrasonography (CEUS) has also been applied to polymyositis and dermatomyositis to assess for vascularity. A study by Weber et al. using replenishment kinetics of microbubbles to measure blood flow, local blood volume, and blood flow velocity showed an increased perfusion in those with edema on MRI and histologically confirmed disease [54]. CEUS blood flow was the best measure and had a sensitivity and specificity of 73% and 91% for a diagnosis of myositis when comparing with histologically confirmed disease. This same group also showed that on follow-up, blood flow parameters decreased in parallel with improvement in disease related parameters [55].

Elastography techniques, as a measure of tissue elasticity or stiffness, are promising for an evaluation of muscle quality in myositis. Earlier studies using strain elastography have shown an increased tissue stiffness in patients with active myositis compared to healthy controls [56, 57]. However no association with abnormal elastography was seen with either MRI or clinically active disease in a study in pediatric patients [58]. Shear wave elastography (SWE), which is a newer generation technique that does not depend on external compression efforts, was recently used to evaluate diseased muscle in IBM [59•]. Lower muscle stiffness was seen to be associated with more severe weakness, and the technique was found to be feasible and reliable (satisfactory within-day and moderate between-day reliability). In another study looking at 23 active myositis patients and comparing this with healthy controls, reduced muscle stiffness was also detected particularly at the thighs [60]. SWE was significantly associated with muscle weakness and MRI signs of edema and atrophy, but not with fat infiltration, CPK levels, or disease duration. Further studies will need to parse out the difference in tissue elasticity between edema and fat replacement and whether this may be a sensitive parameter to follow disease.

## Advantages/Disadvantages of Muscle Ultrasound

The use of muscle US to study muscle diseases has many advantages as demonstrated above. In addition, US can be combined with EMG and muscle biopsies to provide more targeted acquisition of tissue [61]. Muscle skeletal architecture can be assessed non-invasively even in patients where weakness or pain precludes their cooperation [62]. As the technique is well-tolerated even in children and has no contraindications, it allows for repeated measurements of any muscle accessible on US including facial muscles [63, 64].

Some challenges do exist, however, which hamper the widespread utilization of this technique in muscle disease and limit its current use to specialty centers. This includes its operator dependence, subjectivity, and inadequate knowledge of relevant anatomy and appearance of normal and pathological conditions. While quantitative measurements can reduce these issues, there is the difficulty of comparing quantitative measurements between different machines – thus making multi-center studies difficult [65]. Therefore, efforts underway are expected to greatly aid this field in the coming years and include the development of muscle specific systems [66], and new techniques for image analysis that are less dependent on measures of echogenicity [51•, 52].

For myositis in particular, US presents some unique challenges and opportunities. As muscle edema is an important lesion for this disease group and can improve with immunosuppressive treatment, its accurate detection is of primary importance. Further studies systematically characterizing the appearance of muscle edema by correlation with MRI are needed and will greatly advance the field. In chronic forms like IBM where echogenicity is reflective of advancing disease, prospective and longitudinal studies will help to understand its role in follow-up evaluations and potentially in therapeutic trials. Skin and subcutaneous involvement with inflammation or calcinosis also present well for investigation by US, and have not yet been fully explored as part of an overall evaluation of myositis.

## Conclusion

In summary, muscle US is a highly adaptable technique for evaluation of muscle conditions. Scanning protocols can be created to tailor to the disease being studied, with both qualitative and quantitative assessments. As more experience is gained regarding the use of US in inflammatory conditions, we expect that evidence for its utility in muscle disease will continue to increase as it finds its place in the diagnostic toolbox of the rheumatologist.

## Data Availability

Not applicable
